# Humor Improves Women’s but Impairs Men’s Iowa Gambling Task Performance

**DOI:** 10.3389/fpsyg.2019.02538

**Published:** 2019-11-15

**Authors:** Jorge Flores-Torres, Lydia Gómez-Pérez, Kateri McRae, Vladimir López, Ivan Rubio, Eugenio Rodríguez

**Affiliations:** ^1^Escuela de Psicología, Facultad de Ciencias Sociales, Pontificia Universidad Católica de Chile, Santiago, Chile; ^2^Department of Psychology, College of Arts, Humanities and Social Sciences, University of Denver, Denver, CO, United States

**Keywords:** decision-making, humor, gender differences, Iowa gambling task, cognitive control

## Abstract

The Iowa Gambling Task (IGT) is a popular method for examining real-life decision-making. Research has shown gender related differences in performance, in that men consistently outperform women. It has been suggested that these performance differences are related to decreased emotional control in women compared to men. Given the likely role of emotion in these gender differences, in the present study, we examine the effect of a humor induction on IGT performance and whether the effect of humor is moderated by gender. IGT performance and parameters from the Expectancy Valence Model (EVM) were measured in 68 university students (34 men; mean age 22.02, SD = 4.3 and 34 women; mean age 22.3, SD = 4.1) during a 100 trial-IGT task. Participants were exposed to a brief video before each of the IGT decisions available; one half of the samples (17 men and 17 women) was exposed to 100 humor videos, while the other half was exposed to 100 non-humor videos during the task. We observed a significant interaction between gender and humor, such that under humor, women’s performance during the last block (trials 80–100) improved (compared to women under non-humor), whereas men’s performance during the last block was worse (compared to men under non-humor). Consistent with previous work, under non-humor, men outperformed women in the last block. Lastly, our EVM results show that humor impacts the learning mechanisms of decision-making differently in men and women. Humor impaired men’s ability to acquire knowledge about the payoff structure of the decks, and as a consequence, they were stuck in suboptimal performance. On the other hand, humor facilitated women’s ability to explore and to learn from experience, improving performance. These findings deepen our understanding of the mechanisms underlying IGT decision-making and differential effects of humor in men and women.

## Introduction

In addition to performing other computations, the brain can be considered a decision-making device, such that perceptual, mnemonic, and motor capabilities evolved to support the decisions that lead to adaptive actions (Gazzaniga, 2014). One of the most popular tasks to measure decision-making is the Iowa Gambling Task (IGT; Bechara et al., 1994; Hooper et al., 2004), which mimics real-life decision-making in several ways. During the IGT, participants choose from four decks of cards. Each deck yields a fixed, predetermined proportion of monetary punishments and rewards. Participants learn, through exploration, which deck (or decks) will allow them to maximize their earnings as they play, based on their experience receiving feedback from the decks on each trial. Good total performance results from discriminating between “advantageous” (low risk decks) and “disadvantageous” (high risk decks). While total performance is informative, the Expectancy Valence Model (EVM) allows for the separate characterization of three different candidate mechanisms that each influence performance. Namely, three parameters (“*w*”, “*a*”, and “*c*”) can be computed (Busemeyer and Stout, 2002). The parameter “*w*” indicates the extent to which participants are more motivated by rewards or by punishments. The parameter “*a*” indicates the extent to which participants learn by updating their expected valences (the expected monetary net profit for each deck) with experience, or whether the initial expected valences remain influential. Finally, the parameter “*c*” indicates the extent to which participants use the expected valences to guide their decisions, or whether their choices are random. Therefore, by computing these parameters, we have a more nuanced understanding of individual and group differences in task performance.

The idea that emotions are relevant during IGT learning is well-established (van den Bos et al., 2013). According to the Somatic Marker Hypothesis (Bechara et al., 1996), emotions signal how likely it is to obtain punishment or reward, guiding decision-making in situations of complexity and uncertainty (Bechara and Damasio, 2005). According to this theoretical framework, decision-making depends on two neural systems: emotional and cognitive (Bechara, 2005). The emotional system encompasses the orbitofrontal cortex (OFC), the amygdala, and the ventral striatum/nucleus accumbens (Bechara, 2005). The cognitive system encompasses the dorsolateral prefrontal cortex (dlPFC), the anterior cingulate cortex (ACC), and the dorsal striatum (Bechara, 2005; van den Bos et al., 2013). The emotional system signals the actual or anticipated pain or pleasure of feedback, while the cognitive system allows for control of decisional behavior, often by suppressing the activity of the emotional system during the last blocks of the task (Bechara, 2005; van den Bos et al., 2013).

There is consistent evidence that there are gender differences in IGT performance (Reavis and Overman, 2001; Bolla et al., 2004; Overman et al., 2006; Weller et al., 2010). Research shows that, compared to men, during the IGT, women usually obtain less money and need more trials to consistently choose the advantageous decks (Bolla et al., 2004; Weller et al., 2010). Additionally, neurobiological evidence indicates that compared to men, women show hypoactivation of several brain structures (i.e., OFC, dlPFC, and nucleus accumbens) while solving the IGT (see van den Bos et al., 2013). For instance, women exhibit less activation of right lateral orbitofrontal cortex (l-OFC), instead engaging the left medial orbitofrontal (mOFC); by contrast, men exhibit extensive activation of both the right and the left l-OFC (Bolla et al., 2004). The m-OFC seems to be implicated in processing regular patterns, comparing options which are close in reward value, with a focus on more immediate rewards. In contrast, the I-OFC has been implicated in processing with irregular patterns, valuing, and adjusting choice behavior as contingencies change with a focus on long-term rewards (Byrnes et al., 1999; Hooper et al., 2004; Frank and Claus, 2006). Therefore, gender differences in OFC activation may indicate that, women would focus more on immediate and regular patterns of choices than on irregular ones; by contrast, men would focus on irregular patterns of choices. This could help explain why women may need more trials than men before they adjust their choice behavior (Overman et al., 2011). Additionally, gender differences in the activation of the ventral striatum/nucleus accumbens during the IGT have also been reported (van den Bos et al., 2012; Van Hasselt et al., 2012). Namely, women show hypoactivation of ventral striatum/nucleus accumbens than men during IGT. The ventral striatum/nucleus accumbens is involved in reward learning, especially during the initial blocks of the task (Bechara, 2005). However, as the task progresses, activation of brain structures sensitive to short-term reward decreases, and brain structures associated to long-term advantageous choices control the task (Bechara, 2005).

There is evidence suggesting that brain structures hypoactivated by women during the IGT are functionally connected (see Azim et al., 2005; van den Bos et al., 2013) and involved in the transition from emotional to cognitive control during the IGT (Gray et al., 2002; Ueda et al., 2003; Tanaka et al., 2007, 2016; Doya, 2008; Homberg et al., 2008). Therefore, women’s IGT performance could benefit from any intervention that increases the activation of these key brain structures and, as a result, strengthens the transition from emotional to cognitive control. We suggest that humor could be a good candidate.

Humor is a pleasurable and enjoyable experience associated with reward activity (Mobbs et al., 2003) that most scientists consider an extension of social play (Martin, 2007). The difference between humor and other types of social play is that, when we are experiencing humor, we are not playing with physical objects but with concepts and ideas that resolve seeming contradictions (Gervais and Wilson, 2005). Humorous “resolutions” often do not actually make sense in the real world, and they are a way of playing creatively with the cognitive mechanisms that we normally use in more serious contexts (Forabosco, 1992).

Women seem to hyperactivate some humor-related brain structures compared to men (Weinberger, 1993; Diekamp et al., 2002; Gray et al., 2002; Ueda et al., 2003; Azim et al., 2005). Moreover, some of the brain structures than women activate more than men when processing humor are the same structures that seem to be hypoactivated when women perform the IGT. For instance, there is evidence that under humorous conditions, women show greater activation of nucleus accumbens and recruit left prefrontal cortex more strongly than men, suggesting a larger reward network response (Azim et al., 2005). As such, it has been suggested that humor may increase women’s cognitive control through a dopaminergic pathway (Weinberger, 1993; Gray et al., 2002; Ueda et al., 2003; Azim et al., 2005). Generally, reward-related dopamine is likely to exert a multi-faceted influence upon decision-making, through the activity of its forward afferents along the mesolimbic, striatal, and cortical pathways, with the nucleus accumbens playing a pivotal role in action selection (Everitt and Robbins, 2005). According to psychopharmacology research, dopamine activity can influence decision-making by modulating what is learned about the value of an outcome (Lidow et al., 1991; Goldman-Rakic et al., 1992; Frank et al., 2007; Schwartenbeck et al., 2014). If so, humor may facilitate reward learning in women, but not necessarily in men. However, this hypothesis remains to be tested.

The main objective of the present study was to examine the effect of humor on IGT performance and whether the effect of humor is moderated by gender. Humor was induced by asking participants in an experimental condition to watch humorous videos interspersed with the IGT. Participants under a control condition watched non-humor videos instead. Informed by previous research, we hypothesized that humor would increase women’s IGT performance. Therefore, we predicted that women in the humor condition would choose more cards from advantageous decks than women in the non-humor condition, specifically toward the end of the task (namely during blocks three, four, and five), because we hypothesize that humor will facilitate the transition from emotional control to cognitive control (Bechara, 2005). By contrast, humor should not affect men’s IGT performance. Therefore, we did not expect to find statistically significant differences between men in the humor and non-humor conditions in the number of cards chosen from advantageous decks. As mentioned above, in previous studies, men have achieved higher IGT performance than women (Bolla et al., 2004; Weller et al., 2010). These gender differences occur only during the last blocks of the task (namely during blocks three, four, and five; Reavis and Overman, 2001; Overman et al., 2006; Weller et al., 2010). Therefore, we expect to find that the women in the non-humor condition will choose less advantageous deck cards than the men in the non-humor condition, specifically, at blocks three, four, and five. Finally, the effect of humor on the processes underlying IGT decision-making will also be explored from the EVM perspective, which, to our knowledge, has not been previously done and could potentially help us understand gender differences in performance.

## Method

### Participants

Inclusion criteria for participation were (1) being an undergraduate student at the Pontificia Universidad Católica de Chile, (2) being older than 18 years old, (2) speaking Spanish, and (3) having normal or corrected-to-normal vision^1^. Exclusion criteria were (1) reporting severe depressive symptomatology according to the Self-Report Questionnaire (SRQ) (Harding et al., 1980; Vielma et al., 1994), (2) reporting the presence a neurological disorder, (3) reporting a history of drug abuse, and (4) reporting having consumed alcohol, caffeine or drugs 24 h before participating in the experimental task.

In order to estimate the sample size, we used the software G* Power 3.1 with parameters for large effect size *F*s (0.40), a probability error *α* (0.05), and a statistical power of (0.80), which leads to a minimum of 52 participants (26 women and 26 men). Nonetheless, when we reached the sample size of 26 women, there were not still enough men enrolled, so we decided to continue the recruitment till we get an equivalent number of men and women. As such, 72 participants (37 women and 35 men) completed the study. Data from four of these participants (one man and three women) were excluded from the analyses because they did not fulfill all participation criteria. Namely, one of these participants reported having consumed drugs before the experiments and three reported severe depressive symptomatology according to the SRQ. Therefore, we finally analyzed the data of 68 participants (34 men; mean age 22.02, SD = 4.3 and 34 women; mean age 22.3, SD = 4.1).

### Questionnaires

The Self-Report Questionnaire (Harding et al., 1980) was used to assess depressive symptomatology. It consists of 25 yes/no questions. The SRQ has been validated for the Chilean population (Vielma et al., 1994). Subjects scoring higher than 11 points or answering affirmatively questions 21–25 (elevated probability of depressive symptomatology) were not included in the study sample, as depression has shown to affect decision-making (Dalgleish et al., 2004; Battersby et al., 2006).

The State-Trait Anxiety Inventory (STAI) (Spielberger, 1970) was used to assess anxiety symptoms. It consists of 40 questions divided into two subscales: state anxiety (SA) and trait anxiety (TA). The STAI has been validated for Chilean population (Vera-Villarroel et al., 2007). We assessed this variable because higher trait anxiety scores are related to impairments in decision-making and could potentially affect our results (Miu et al., 2008).

#### Humorous and Non-humorous Videos

To induce humor, we selected 200 videos (100 humorous and 100 non-humorous) from 240 public access videos (120 humorous and 120 non-humorous) available at www.youtube.com. Selection criteria were the presence or absence of humor in ecological situations [i.e., humorous videos depicted situations with non-sensical or with incongruity resolution structure while non-humor videos depicted mundane situations in which nothing of any emotional impact occurred (e.g., mowing the lawn, walking down the street)], and an adequate duration of stimuli to present a video before each decision (raw video mean duration = 12.84 s; video SD = 5.81 s). The total 240 videos were presented in a randomized order to 50 subjects (25 men and 25 women) who were not part of the present sample. We asked them to rate the videos using a humor scale ranging from 0 to 10 points (0 “not humorous at all”; 10 “the most humorous thing ever”). We eliminated all the videos that showed significant differences in ratings between men and women, as well as those less than three standard deviations away from the mean of the opposite condition, which resulted in the elimination of 40 videos (20 humorous and 20 non-humorous). The final video selection consisted of 100 humorous and 100 non-humorous videos. Men (*humor*: mean = 4.18, SD = 0.51; *non-humor*: mean = 1.50, SD = 0.25). Women (*humor*: mean = 4.24, SD = 0.46; *non-humor*: mean = 1.51, SD = 0.23). To examine whether there were statistically significant differences in the video ratings, we conducted a two-way factorial ANOVA (Gender × Video type [humor/non-humor]). Results showed a main effect of video type (*F*_1,198_ = 220, *p* < 0.001, *η*^2^ = 0.917), indicating that humorous videos were rated as significantly more humorous than non-humorous videos. Neither the main effect of Gender nor the interaction (Gender × Video type) was statistically significant. Humorous videos were assigned to the experimental group and non-humorous videos to the control group. The mean duration of the final selection of the videos was 12.91 (SD = 5.89) and 10.41 s (SD = 5.03) for the humorous and the non-humorous videos, respectively^2^.

#### The Iowa Gambling Task

The IGT was designed as a realistic decision-making task (Bechara et al., 1994; Hooper et al., 2004). On each trial, participants choose a card from one of four card decks (A, B, C, and D). After each choice, participants are rewarded with virtual money (reward) or punished with a loss of virtual money (punishment). Participants must learn as they play which are the advantageous and disadvantageous decks to solve the task and maximize earnings. Participants can change decks at will; however, they are warned that some decks are worse than others in terms of total payment, and that the win/loss proportions and amounts stay fixed within each deck. Likewise, they are informed that the goal is to win as much money as they can, or to avoid losing money as much as possible.

Card decks A and B are monetarily risky/disadvantageous, and C and D are monetarily safe/advantageous. Card decks A and B are associated with large, immediate rewards (e.g., $100) but continuing to select from these decks results in accumulating less profit, or loss, because of occasional, large monetary punishments. Choosing from card decks A and B leads to a net loss of $250 during the first 10 trials. By contrast, card decks C and D are associated with small immediate rewards (e.g., $50) but with small monetary punishments. Continuing to select from these decks results in accumulating more profit, and choosing from decks C and D leads to a net gain of $250 during the first 10 trials.

An outcome score was calculated by subtracting the total number of cards selected from the disadvantageous decks (A + B) from the total number of cards selected. The remaining cards are from the advantageous decks (C + D) for each of the 5 sets of 20 choices, called blocks (Bolla et al., 2004; Overman et al., 2006; Weller et al., 2010).

### Procedure

The Ethics Committee of the Pontificia Universidad Católica de Chile (PUC) approved the study. All experiments were performed at the Neuro-dynamic Laboratory of the School of Psychology of the PUC. We recruited participants for the study through an advertisement published in the PUC student website. Those interested in participation were informed about the inclusion and exclusion criteria and provided with more study details *via* email. If they reported that they met the inclusion criteria, we finally invited them to come to the lab.

In-lab session, first, we provided participants with more details about the study and completed the informed consent process. Next, participants completed a battery of questionnaires comprised of the SRQ and STAI-t. Then, they sat down in a comfortable chair in front of a computer screen and completed the IGT. Task instructions were presented in writing on the computer screen. The distance from participant’s eyes to computer screen was 60 cm, visual angle 4.7°. Study duration was approximately 1 h. Participants received one movie ticket in compensation for participation.

Each trial began with the word “*video*,” which appeared on the screen for 1,500 ms. Then the video itself appeared, followed by a decision-making trial. During these trials, participants saw four deck options (labeled A, B, C, and D) and chose one by clicking on the deck with a USB mouse. When participants selected a deck, its perimeter lit up in red. After that, the screen changed to black for 200 ms, after which, the feedback appeared for 2,000 ms. Feedback could be a win (e.g., you won +100) or a win and a loss (e.g., you won 100, but lost −50). Each card’s feedback depended on the probabilities according to IGT manual (Bechara, 2007). During the screen showing the four deck options, on the central superior area of the screen, two bars appeared. A green bar showed cumulative wins and losses and a red bar represented the amount of money they owed (all participants started the task with $2,000 CLP virtual money). After feedback, these bars automatically updated according to the feedback on that trial. We emphasized to participants that positions and deck contingencies were fixed during the whole task, that they could change decks at will, and that there was no association whatsoever between the videos and the decks. Participants had no specific information about how to solve the task, nor did they know how long it would take. Participants completed 100 videos and 100 trials (divided into 5 blocks of 20 trials each).

### Calculation of Expectancy Valence Model Parameter

The EVM is a reinforced learning model. It produces three cognitive processes parameters, “*w*”, “*a*”, and “*c*”. According to Wetzels et al., 2010, the model assumes that, after selecting a card from deck *k, k* ϵ {1, 2, 3, 4} on trial *t*, participants calculate the resulting net profit or valence. This valence *v_k_* is a combination of the experienced reward W(*t*) and the experienced loss L(*t*):

$$v_{k}\;\left(t\right)=\left(1-w\right)\;W\left(t\right)+w\;.\;L\left(t\right)$$ (1)

This equation uses the EVM parameter “*w*,” which provides information about whether participants pay more attention to, or are more motivated by, rewards compared to punishments. Values of “*w*” range between 0 and 1. Values lower than 0.50 are indicative of being relatively more motivated by rewards than by punishments, whereas values higher than 0.50 are indicative of being relatively more motivated by punishments than by rewards, and values equal to 0.50 are indicative of being equally motivated by rewards and punishments (Wetzels et al., 2010).

Based on the sequence of valences *v_k_* experienced previously, the participants form an expectation *Ev_k_* of the valence for deck *k.* Learning occurs when new feedback changes the value of the expected valence *Ev_k_*. In a given time *t*, if the experienced valence differs from the expected one, then the value *Ev_k_* needs to be adjusted. The way the value is adjusted is given by the following equation:

$$Ev_{k}\;\left(t+1\right)=Ev_{k}\;\left(t\right)+\alpha \;.\;\left(v_{k}\left(t\right)-Ev_{k}\left(t\right)\right)$$ (2)

In this equation, the updating rate *α* ϵ [0, 1] determines the impact of recently experienced valences. Opting for the deck with the highest expected valence is a “greedy” strategy that in the long run can lead to a suboptimal solution, given it involves little exploration. To ensure initial deck exploration from the participants, an additional equation is added to the model. The equation is a standard reinforcement learning method called softmax selection or Boltzman exploration:

$$\mathrm{Pr}\left[S_{k}\;\left(t+1\right)\right]=\frac{\exp\left(\theta \left(t\right)Ev_{k}\right)}{\sum_{j=1}^{4}\exp\left(\theta \left(t\right)Ev_{j}\right)}$$ (3)

In this equation, $\frac{1}{\theta \left(t\right)}$ is the “temperature” at the trial *t*, and Pr(*S_k_*) is the probability of selecting a card from deck *k.* Higher temperatures mean more random decisions, which means a higher level of exploration, while lower temperatures mean less exploration, and more exploitation of the decks with higher expected valences. A temperature of zero indicates the participant decides only based on expected valence, choosing the deck with the highest expected valence.

In the EV model, the temperature changes, given the number of observations, according to the following formula:

$$\theta \left(t\right)=\;{\left(\frac{t}{10}\right)}^{c}$$ (4)

where “*c*” is the response consistency or sensitivity parameter (also called the exploration parameter). When fitting to data, the parameter is constrained to the interval [−5, 5]. Positive values of “*c*” make response consistency *θ* values increase with the number of observations, which means 1/*θ* values will decrease. This leads to lower “temperatures,” meaning choices are guided more by expected valences. Negative values of “*c*” mean choices will become more and more random as the number of cards selected increases.

Being “*i*” a given participant, the current IGT study calculated participant’s specific parameters “*w_i_*,” “*a_i_*,” and “*c_i_*” by minimizing the sum of the one-step-ahead prediction errors:

$$\sum_{t=1}^{T}-\ln p\left(y_{t}|y^{t-1},\;w_{i},\;a_{i},\;c_{i}\right)$$ (5)

## Data Analysis

Trait anxiety has been shown to affect IGT performance, and women report more trait anxiety than men (Miu et al., 2008); therefore, to examine whether there were differences between the groups in this variable, we first conducted a two-way factorial ANOVA [Gender × Condition (Humor/Non-humor)] in which the dependent variable was trait anxiety. The main effect of Gender, the main effect of Condition, and the interaction were not statistically significant, indicating that there were no significant differences in trait anxiety among groups. Therefore, this variable was not considered in further analyses.

In order to examine the effect of humor on IGT performance and whether the effect of humor differed by Gender, we conducted a three-way ANOVA (Gender × Condition × Blocks) considering as the dependent variable the number of advantageous deck cards chosen (C + D). In addition, in order to explore the effect of humor in the three cognitive latent processes (“*w*,” “*a*,” and “c”) underling decision-making, we performed a two-way (Gender × Condition) MANOVA. Prior to conducting these analyses, we checked normality, linearity, and sphericity assumptions. When needed, outliers were replaced using the mean plus two standard deviations method recommended by Field (2013). In case the sphericity assumption was violated, we used the parameter ε Greenhouse-Geisser to correct for such violations. We applied a Bonferroni correction to *post-hoc* comparisons. Table 1 shows descriptive statistics for IGT performance and Table 2 shows descriptive statistics for Expectancy Valence Model.

**Table 1:** Descriptive statistics for IGT performance.

		Experimental (*n* = 34)	Control (*n* = 34)
		*M* (SD)	*M* (SD)
Men (*n* = 34)	B1	8.12 (2.29)	9.12 (1.93)
	B2	8.41 (2.57)	9.71 (3.33)
	B3	10.06 (1.95)	10.59 (2.40)
	B4	10.71 (2.93)	7.71 (3.74)
	B5	9.47 (1.59)	11.71 (3.90)
Women (*n* = 34)	B1	9.94 (1.68)	10.29 (1.49)
	B2	12.18 (3.97)	10.06 (2.11)
	B3	11.65 (3.77)	9.82 (1.63)
	B4	11.00 (3.55)	11.00 (2.52)
	B5	11.65 (4.43)	9.06 (2.56)

**Table 2:** Descriptive statistics for Expectancy Valence Model.

		Experimental (*n* = 34)	Control (*n* = 34)
		*M* (SD)	*M* (SD)
Men (*n* = 34)	Parameter “*w*”	0.44 (0.41)	0.48 (0.40)
	Parameter “*a*”	0.0003 (0.00039)	0.003 (0.003)
	Parameter “*c*”	−1.4 (1.19)	−0.97 (0.64)
Women (*n* = 34)	Parameter “*w*”	0.08 (0.09)	0.17 (0.21)
	Parameter “*a*”	0.0016 (0.0018)	0.0004 (0.00048)
	Parameter “*c*”	−0.41 (1.21)	−0.39 (1.35)

## Results

### Differences in Iowa Gambling Task Performance

**Figure 1:**
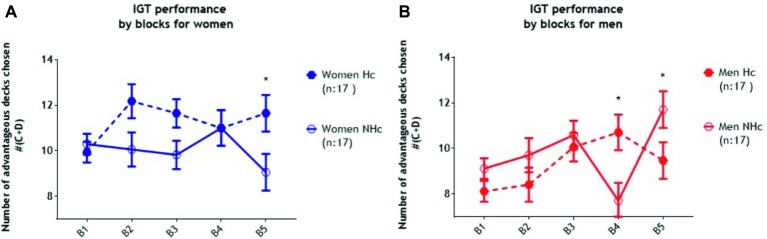
Results for IGT performance under the non-humor condition (NHc: 17 men and 17 women) and the humor condition (Hc: 17 men and 17 women) with standard error of the mean (SEM). The 100 trial-task was divided into 5 blocks of 20 trials each. **(A)** Analysis for Blocks × Condition in women revealed significant differences for Block 5 benefiting Hc over NHc. **(B)** Analysis for Blocks × Condition in men revealed significant differences for Block 4 benefiting Hc over NHc, and during Block 5 inverting the relationship, benefiting NHc over Hc.

**Figure 2:**
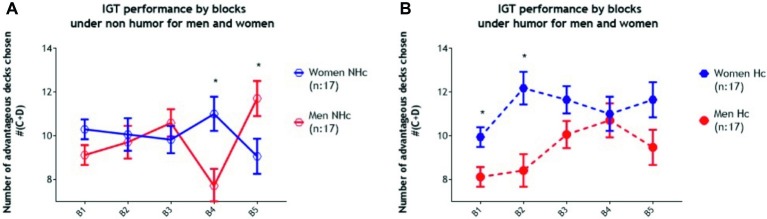
Results for IGT performance under the non-humor condition (NHc: 17 men and 17 women) and the humor condition (Hc: 17 men and 17 women) with standard error of the mean (SEM). The 100 trial-task was divided into 5 blocks of 20 trials each. **(A)** Analysis for Blocks × Condition under NHc revealed significant differences during Block 4, benefiting women over men, and Block 5, benefiting men over women. **(B)** Analysis for Blocks × Condition under Hc revealed significant differences during Block 1 and 2, benefiting women over men.

The result of the three-way ANOVA (Condition × Gender × Block) revealed a significant main effect of Gender (*F*_1, 64_ = 4.35, *p* = 0.04, *partial η^2^* = 0.06) indicating that men chose fewer cards from advantageous decks than women overall. There was a significant main effect of Block (*F*_3.39, 217.07_ = 3.42, *p* = 0.014, *partial η^2^* = 0.05), indicating that participants improved their performance across the task. Neither the main effect of Condition (*F*_1, 64_ = 0.60, *p* = 0.44, *partial η^2^* = 0.00) nor the interaction of Gender × Condition (*F*_1, 64_ = 2.41, *p* = 0.13, *partial η^2^* = 0.04) were statistically significant. However, the interaction of Gender × Block was statistically significant (*F*_3.39, 217.07_ = 9.05, *p* < 0.001, *partial η^2^* = 0.12), indicating that men chose fewer cards from advantageous decks than women at block four (*t* = −2.98, *p* < 0.01, *partial η^2^* = 0.12) but more from advantageous decks at block five (*t* = 2.33, *p* = 0.02, *partial η^2^* = 0.08) collapsing across condition. Critically, the Block × Condition × Gender interaction was statistically significant (*F*_3.39, 217.07_ = 9.05, *p* < 0.001, *partial η^2^* = 0.12) indicating that women in the experimental (humor) condition selected more cards from the advantageous decks than women in the control (non-humor) condition by block five (*t* = 2.09, *p* = 0.04, *partial η^2^* = 0.06). See Figure 1A. The situation for the men was very different. Men in the humor condition chose more cards from advantageous decks than those in the non-humor condition during block four (*t* = 2.60, *p* = 0.01, *partial η^2^* = 0.18), but during block five, the situation reversed completely, and men in the non-humor condition chose more advantageous deck cards than those in the humor condition (*t* = −2.19, *p* = 0.04, *partial η^2^* = 0.13) (see Figure 1B). Directly comparing men and women, in the non-humor condition, men chose fewer cards from the advantageous decks than women at block four (*t* = −3.01, *p* < 0.01, *partial η^2^* = 0.22), but at block five, men in the non-humor condition chose more cards from advantageous decks than women in the non-humor condition (*t* = 2.34, *p* = 0.03, *partial η^2^* = 0.15) (see Figure 2A). Furthermore, women in the humor condition chose more cards from advantageous decks than men in the humor condition at block one (*t* = −2.65, *p* < 0.01, *partial η^2^* = 0.18) and block two (*t* = −3.28, *p* < 0.01, *partial η^2^* = 0.25). See Figure 2B.

### Differences in Expectancy Valence Model Parameters

**Figure 3:**
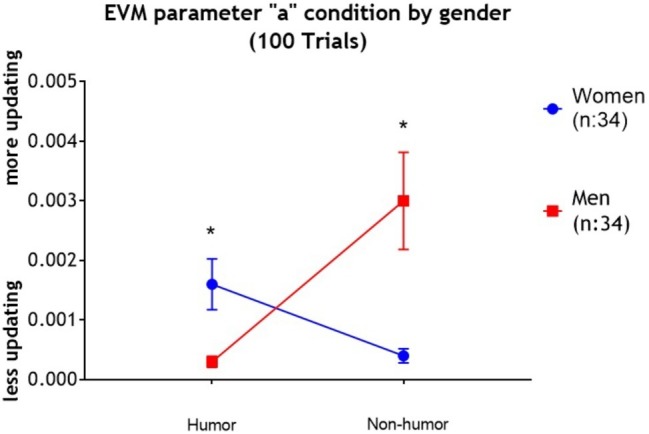
Results for the EVM analysis on parameter “*a*” (updating rate score) with standard error of the mean (SEM). Analyses revealed a significant interaction of Gender × Condition, indicating that women in the humor condition had higher scores than men in the humor condition, and men in the control condition had higher scores than women in the control condition. We also observed that women in the humor condition had higher scores than women in the control condition. Finally, men in the humor condition had lower scores than men in the control condition.

The result of the two-way (Gender × Condition) MANOVA indicated that the multivariate main effect of Gender was statistically significant, Pillai’s Trace *V* = 0.264, *F*(3,62) = 7.43, *p* = 0.001, *partial η^2^* = 0.264. Univariate analyses showed that collapsing across condition, compared to women, men had higher “*w*” scores *F*(1,64) = 19.89, *p* < 0.001, *η^2^* = 0.237, *M*_men_ = 0.458, SD_men_ = 0.399; *M*_women_ = 0.123, SD_women_ = 0.167, and lower “*c*” scores, *F*(1,64) = 8.15, *p* < 0.006, *partial η^2^* = 0.113, *M*_men_ = −1.18, SD_men_ = 0.96; *M*_women_ = −0.4, SD_women_ = 1.2. The multivariate main effect of condition was not statistically significant, Pillai’s Trace *V* = 0.072, *F*(3,62) = 0.161, *p* = 0.196, *partial η^2^* = 0.072. Finally, the multivariate interaction of Gender × Condition was statistically significant, Pillai’s Trace *V* = 0.224, *F*(3,62) = 5.98, *p* = 0.001, *partial η^2^* = 0.224. The results of the univariate ANOVAs showed that the interaction effect was statistically significant only for the parameter “*a*,” *F*(1,64) = 16.95, *p* < 0.001, *partial η^2^* = 0.21 (see Figure 3). Namely, men in the humor condition had significantly lower parameter “*a*” scores than women in the humor condition (*t* = −2.86; *p* < 0.01; *partial η^2^* = 0.20; *M*_men humor_ = 0.0003, SD_men humor_ = 0.0004; *M*_women humor_ = 0.0016, SD_women humor_ = 0.0017). Conversely, men in the non-humor condition had higher parameter “*a*” scores than women in the non-humor condition (*t* = 3.15; *p* < 0.01; *partial η^2^* = 0.24; *M*_men non-humor_ = 0.003, SD_men non-humors_ = 0.003; *M*_women non-humor_ = 0.0004, SD_women non-humor_ = 0.0004). In addition, women in the humor condition had higher parameter “*a*” scores than women in the non-humor condition, (*t* = 2.59; *p* < 0.05; *partial η^2^* = 0.17). For men, the situation was reversed: men in the humor condition had lower parameter “*a*” scores than the men under the non-humor condition (*t* = −3.29; *p* < 0.01; *partial η^2^* = 0.25).

## Discussion

The main purpose of the present study was to examine the effect of humor on IGT performance, and whether the effect of humor on IGT was moderated by gender. We expected that humor would increase women’s, but not men’s IGT performance, specifically during the last blocks of the task. In line with our hypothesis, we found that women exposed to humorous videos outperformed women exposed to non-humorous videos at the end of the task (block five). The effect size of humor was medium to large (*d* = 0.72). Consistent with this, we found that women in the humor condition had higher parameter “*a*” scores than women in the control condition (reflecting an increase in memory/learning processes). The effect size of humor on parameter “*a*” was large (*d* = 0.91).

Contrary to our hypothesis, we did find an effect of humor on men’s IGT performance at the end of the task (block five), unlike women, men in the humor condition underperformed on the task compared with men in the control condition. The effect size of humor on men’s IGT performance was medium to large (*d* = 0.75). We found it striking that humor improved women’s IGT performance at block five, but impaired men’s performance at this very same block. We also found significant differences between men in the humor condition and men in the control condition at block four, with men in the humor performing better than men in the non-humor condition. The effect size was large (*d* = 0.89). Consistent with this, men in the humor condition had lower parameter “*a*” scores than men in the control condition (reflecting a decrease in memory/learning processes). The effect of humor on men’s parameter “*a*” was large (*d* = 1.26).

Our results strongly imply that humor is beneficial for decision-making, but only in women. IGT research has shown that during the last blocks of the task, performance depends on a cognitive brain system, which allows cognitive control of long-term decisional behavior and suppresses the activity of an emotional system that triggers impulsive short-sighted decisions (Bechara, 2005; van den Bos et al., 2012, 2013). In order to successfully solve the task, participants need to exert top-down control to stop focusing on regular and immediate rewards, and pay attention to more irregular and long-term rewards (Bechara, 2005; van den Bos et al., 2013). We suggest that humor may influence women’s decision-making by facilitating cognitive control during the last block of the IGT, helping women to choose cards from decks that provide long-term rewards. According to neurobiological evidence, changing to long-term decisions requires an increase in dlPFC activity (Knoch et al., 2006; Fecteau et al., 2007), which is usually hypoactivated in women during the IGT, and may be related to their difficulty exerting consistent cognitive control as the task unfolds (van den Bos et al., 2013). Consistent with our results, humor has been shown to indirectly increase dlPFC activity among women, *via* nucleus accumbens activity (Gray et al., 2002; Ueda et al., 2003; Azim et al., 2005; Tanaka et al., 2007; Doya, 2008; Homberg et al., 2008; Tanaka et al., 2016). Thus, it is possible that humor enhances cognitive control by increasing the activity of these areas during the IGT. Nevertheless, our behavioral study cannot directly measure brain activity, so further research is needed to support this hypothesized neural mechanism by which humor improves IGT performance in women.

Another possibility is that humor may have influenced decision-making by modulating the value of the expected valences, or “updating rate,” during the task (parameter “*a*”). In terms of memory/learning, parameter “*a*” reflects the impact of recently experienced valences. Small values of parameter “*a*” are indicative of slow changes, weak recency effects, long associative memories, and slow forgetting during the task (Wetzels et al., 2010). We found that women in the experimental condition had higher parameter “*a*” scores than women in the control condition. This indicates that they demonstrated more efficient memory/learning processes, showing more deck exploration and integrating feedback information as a possible expected value to a greater extent. They explored different decks other than A and B more frequently than women in the control condition, which therefore may have helped them form a better representation of the long-term advantages of decks C and D. In fact, women in the control group showed a mean value of parameter “*a*” of 0.0004, indicating they seem to explore almost never, which reflects a state of no knowledge about the payoff structure of the decks (Humphries et al., 2015).

It is difficult to explain why we did not observe a significant effect of humor on IGT performance in women during blocks three and four. One possibility is that the strength of the humor manipulation grows over time. In our study, participants had a total of 12 min of humor induction and approximately 8 min of IGT decisions, adding to 20 min total. One study showed that after 30–40 min, humor seems to have stronger physiological effects (Weisenberg et al., 1998). Therefore, we presume that, if the number of IGT trials were increased, differences in performance between women in the humor and control conditions would be systematically found from block 5 onwards. Future studies using more trials and/or longer periods of emotional induction are needed.

Contrary to our hypothesis, we found statistically significant differences in the performance of men in the humor and control conditions. Men in the humor condition performed better than men in the control condition during block four, but during block five the situation reversed, and men in the control condition performed better than men in the humor condition. Additionally, men in the humor condition had lower parameter “*a*” (updating rate) scores than men in the control condition.

A closer inspection of the data reveals that during blocks 1–3, men in both groups show slow learning from choosing very few C and D deck cards (mean of 8 out of 20) in block 1 and 2, and a mean close to 10 in block 3, decisions that were nearly random. As such, among men generally, the knowledge of the task was likely extremely low during the first three blocks. According to Damasio (2003) participants need to acquire implicit knowledge of the value structure of the decks in order to later on ensure advantageous behavior. Near random choices indicate a failure in learning the differential value structure of the decks. Additionally, research on IGT supports the notion that beyond an emotional hunch, a minimum level of explicit knowledge about the value of the decks is necessary to generate a hypothesis about which deck/s are necessary to maximize performance (Maia and McClelland, 2004). In order to reverse our bad choices, we at least need to have the notion that we are doing it badly (explicit knowledge, Maia and McClelland, 2004), so “taking a risk” is a good option when we already know that doing the same is equal to or worse than doing something different. Under these circumstances, prospect theory (Kahneman and Tversky, 1979) predicts that we will risk when we know that losing is very probable. The pronounced decrease in the number of cards chosen from advantageous decks from block three to block four among men in the control condition, and the abrupt increase we observed from block four to block five can be interpreted in the light of this theoretical framework.

A completely different scenario is observed in men in the humor condition. Men in the humor condition show very little risk-taking, and very low explicit knowledge, as evidenced by near-chance deck selection in blocks 4 and 5. The EVM results support our interpretation, as men in the humor condition had extremely low parameter “*a*” scores, indicating zero knowledge about the value structure of the decks, while those under the non-humor control condition had significantly higher parameter “*a*” scores, indicating more learning about the value structure of the decks over time.

The detrimental effect of humor on IGT decision-making performance in men, to our knowledge, has not been previously reported. As we formerly stated, we suggest that the men under humor could not form the body of knowledge of the decks’ value, which is necessary to explicitly form hypotheses and strategize to maximize earnings. One could possibly think that this decisional behavior reflects the emotional system predominance over the task. Nevertheless, if the emotional system were “in control” of the decisional behavior of men in the humor condition, we would expect that choices with the highest expected valence were chosen (choices that provide highest reward value), leading to a focus mostly on immediate rewards (A + B deck choices). But as we previously mentioned, their choices were instead mostly random, indicating a state of no knowledge. Using the somatic marker hypothesis as an explanatory framework, it is expected that non-conscious autonomic responses or emotion-based biasing signals, precede explicit insight on the IGT decisions (Damasio, 2003). According to this, what probably happened is that humor interfered with the emotional signals necessary to form “hunches,” or implicit knowledge of the value structure of the decks, so as the task progresses, they never really form explicit knowledge about the decks, producing as a result random choice through the whole task. Regrettably, our behavioral study does not provide data about the neural correlates of attention while participants solve the task. Thus, more research about the differential neural mechanisms by which emotion impacts decision-making in men and women is needed.

Previous studies have found that, compared to men, women usually make poorer IGT choices, and need more trials to solve the task (Bolla et al., 2004; Weller et al., 2010). In line with our hypothesis, at the end of the task, men in the non-humor condition chose more advantageous deck cards than women in the non-humor condition. However, contrary to our hypothesis, at block four women chose more advantageous deck cards than men (in the non-humor condition). No differences were found among men and women at block three. Differences found between women and men at block four and five may be due to the abovementioned implementation of risk by men, which possibly help them to perform better by the end of the task.

Finally, we expected humor would cancel out typical gender differences in IGT performance during the last blocks. In line with this hypothesis, we found no statistical differences between men and women in the humor condition during blocks three, four and five. Therefore, the gender differences observed in the non-humor condition during block five were not present in the humor condition. This was the combined result of two effects: men in the humor condition showed decreased performance in block five, and women in the humor condition showed improved performance in the same block.

Our study differed from previous studies in the use of videos that interspersed each decision (100 videos for 100 choices). Therefore, our participants needed to split their attention between the videos and the decisions while performing the task. A previous study (Preston et al., 2007) found that men had poorer IGT performance than women when their attention was divided between the IGT and another task. So, the exposure of our participants to a dual-task like paradigm may have had a more negative impact on men than on women. We propose that decreased attention could be the mechanism by which humor impairs men’s performance, which would lead to almost no exploration, slower learning, and poor total choice behavior. Alternatively, humor may have been detrimental to men’s decision-making performance because the funny videos may have decreased their motivation to complete the task in a serious way, by shifting their mindset from a serious (bona-fide performance) to a playful (non-bona-fide mode), devaluing the goal of performing well. In future studies, measures of humor-related states (e.g., the State-Trait-Cheerfulness Inventory, Ruch, 1997) would allow for more complete characterization of the psychological mechanisms at work here.

The present study has some limitations. First, it is an experiment and therefore, its results may not generalize to real-life situations. Additional studies with higher ecological validity in which the effect of humor over real-life decision-making is examined are still needed. Second, we did not assess whether the effect of humor on decision-making was affected by the characteristics (content or structure) of the videos used. Studies exploring whether the type of humor moderates the relationship between humor on decision-making need to be conducted. Third, research has shown that reactions to humorous stimuli may cover two orthogonal dimensions, funniness, and aversiveness (see Ruch and Deckers, 1992; Ruch and Rath, 1993; Heintz, 2019). In our study, we controlled for gender differences in the subjective funniness of the videos, but we did not control for potential gender differences in the subjective aversiveness of the videos. Therefore, our results may have been affected by this extraneous, unmeasured variable. Fourth, it has been previously reported that men prefer sexual (Thorne et al., 1983), aggressive (Brodzinsky et al., 1981; Crawford, 1989; Herzog and Hager, 1995) or dark humor (Aillaud and Piolat, 2012; Martin and Ford, 2018) more than women, therefore, we did not include videos with these types of humor. Our results cannot be generalized to dark humor or to stimuli that are sexual or violent. Fifth, all participants were university students, and the results may not generalize to other samples. Sixth, it may have been interesting to include measurements of positive and negative emotions or attention allocation during the task to examine potential emotional and cognitive mechanisms underlying the effects of humor on IGT performance. Future studies including other behavioral and neurophysiological variables (e.g., ERPs, the PANAS, etc.) are recommended. Seventh, unfortunately, we did not collect information regarding specific emotions (other than humor) evoked by the non-humorous videos. Therefore, we cannot be completely sure that they did not evoke in the participant some type of emotion that could affect our results (e.g., boredom). As such, the results of the present study should be taken with caution and replicated in future studies in which the specific emotions elicited by the videos are collected during the task. Eighth, some preliminary evidence suggests that IGT performance may be affected by stress, especially among women (Preston et al., 2007; van den Bos et al., 2009). It is possible that women experience higher stress during the IGT than men, and that this could explain gender differences in performance. Unfortunately, we did not assess stress during the task. Replication studies in which the influence of stress and other positive and negative feelings are controlled during the task should be conducted in the future. It may be also interesting to know whether similar effects can be produced by inducing other positive emotions and by using methods other than videos. In spite of this, our study has several important strengths. First, this is the first study evaluating the effect of humor on the IGT, and whether that effect is moderated by gender. Additionally, to our knowledge, this is also the first study that uses both IGT decision-making performance evaluation and EVM together to interpret results. Our findings contribute to better understanding the cognitive mechanisms underlying decision-making in men and women. Furthermore, unlike previous studies measuring emotional effects on the IGT, it takes simultaneously gender and humor into account, providing a more complete picture of decision-making, and showing differences that may remain hidden when the moderator of gender is not considered. Also, previous studies involving induction of positive and negative emotions during the IGT have used a single stimulus, of short duration (i.e., a happy or sad video of 2.5 min before the task), without taking into account that this period of time may not be enough to induce a lasting emotional effect across the whole task. We presented the stimulus throughout the task and for longer periods of time, allowing for slow emotional changes in participants as the task progressed.

In conclusion, humor impaired men’s and improved women’s decision-making performance. These differences may be due to gender differences in humor processing and in how men and women efficiently allocate attentional resources in complex scenarios; however, the neural mechanisms underlying these differences remain unclear. Future studies exploring differential brain mechanisms of the effect of humor on decision-making in men and women by means of brain exploration techniques such as electroencephalography and/or fMRI are needed.

## Data Availability Statement

The datasets generated for this study are available on request to the corresponding author.

## Ethics Statement

The studies involving human participants were reviewed and approved by The Ethics Committee of the Pontificia Universidad Católica de Chile (PUC). The patients/participants provided their written informed consent to participate in this study.

## Author Contributions

JF-T contributed in the design, implementation, data analysis and data interpretation of the study. LG-P and IR contributed to the analysis of the data. LG-P, KM, VL, and ER contributed to the interpretation of the data, revised the manuscript critically for important intellectual content, and approved the final version of the manuscript. JF wrote the first draft. LG-P and KM modified the final draft of the article.

### Conflict of Interest

The authors declare that the research was conducted in the absence of any commercial or financial relationships that could be construed as a potential conflict of interest.

## Footnotes

^1^In the present study we did not measure separately Sex and Gender. We will assume participants responses about their sexual identity refer to gender (i.e., men and women) instead of sex (male and female). Data regarding the participants specific gender identities were not collected.

^2^All videos used in the current study are available at the following link: https://www.dropbox.com/sh/qc8n4f2j6v594xs/AABcN7cnh_W-QY3s9KCdpiRHa?dl=0
